# Ni Supported on Hollow CeO_2_ Microspheres with Controllable Shell Thickness for Catalytic Dry Reforming of Methane

**DOI:** 10.3390/nano16140868

**Published:** 2026-07-15

**Authors:** Junyi Liu, Hongyu Cui, Tianqi Cao, Chuanhui Zhang

**Affiliations:** 1 Institute of Materials for Energy and Environment, College of Materials Science and Engineering, Qingdao University, Qingdao 266071, China; liujunyi13@qdu.edu.cn (J.L.); cuihongyu@qdu.edu.cn (H.C.); caotianqi@qdu.edu.cn (T.C.); 2 Faculty of Chemical Engineering and Energy Technology, Shanghai Institute of Technology, Shanghai 201418, China

**Keywords:** dry reforming of methane, hollow nanospheres, metal-support interaction, coke resistance, reaction mechanism

## Abstract

CeO_2_ hollow nanospheres were fabricated through a hard template-assisted route, with the shell thickness precisely tuned by varying the usage amount of Ce(NO_3_)_3_·6H_2_O. Ni was subsequently deposited onto the *x*CeO_2_-H (*x* = 6, 8, 10) supports via an incipient wetness impregnation method, affording Ni/6CeO_2_-H, Ni/8CeO_2_-H, and Ni/10CeO_2_-H catalysts, which were evaluated for catalytic dry reforming of methane (DRM). Notably, the Ni/6CeO_2_-H catalyst delivered stable CH_4_ and CO_2_ conversions of 70% and 76%, respectively, with an H_2_/CO molar ratio of 0.81 over a reaction period of 50 h at 750 °C, demonstrating exceptional catalytic stability. Comprehensive characterization revealed that the Ni/6CeO_2_-H catalyst featured stronger metal–support interaction (MSI) and abundant basic sites, which synergistically enhanced CO_2_ adsorption and activation, thereby endowing the catalyst with superior coke resistance. In situ infrared spectroscopy further elucidated the DRM reaction mechanism, revealing that surface –OH groups played a pivotal role in suppressing coke formation and sustaining high reaction efficiency by reacting with carbon precursor species (CH_x_*).

## 1. Introduction

The continuous growth of global industrial activities has led to a steady increase in fossil fuel consumption, making greenhouse gas emissions a major cause of environmental problems. Among all anthropogenic radiative forcing factors, carbon dioxide (CO_2_) and methane (CH_4_) are the two main contributors to climate change [1,2]. Converting these two gases into high-value products is of great importance for reducing global warming and making better use of carbon resources [3,4]. The dry reforming of methane (DRM) reaction converts CO_2_ and CH_4_ directly into syngas (CO + H_2_) with an H_2_/CO ratio close to 1:1. Compared with steam reforming and partial oxidation, this ratio is more suitable for downstream Fischer–Tropsch synthesis to produce hydrocarbons and oxygenates [5,6]. Nickel-based catalyst is widely used as the active component for DRM because of its low cost and high activity, making it a good alternative to noble metal catalysts such as platinum (Pt), rhodium (Rh), and ruthenium (Ru) [7,8]. However, nickel-based catalyst has two main drawbacks: Nickel (Ni) particles tend to sinter and agglomerate at high temperatures, and carbon deposition on the surface covers active sites and causes catalyst deactivation. These two problems are the biggest obstacles to the industrial usage of Ni-based catalyst in low-temperature DRM [9,10]. Therefore, developing Ni-based catalyst with low cost, high activity, and strong resistance to carbon deposition is essential for the commercialization of DRM. In recent years, researchers have devoted significant efforts to studying various catalyst design strategies, encompassing diverse metal-support combinations [11,12], unique catalyst structures [13,14], and the rational utilization of noble metals [15,16]. In the research about DRM catalyst, commonly used support materials include silica (SiO_2_), magnesium oxide (MgO), alumina (Al_2_O_3_), and ceria (CeO_2_). Among them [17], CeO_2_ is regarded as the most promising support material for Ni-based catalysts due to its rich oxygen vacancies and high oxygen storage capacity, holding the potential to achieve both high activity and high stability in DRM reactions [18,19,20,21]. However, conventional supported catalysts have a large exposed area of active metal particles, making it difficult to effectively suppress Ni sintering and carbon deposition growth at high temperatures. In recent years, researchers have carried out extensive design strategy studies on CeO_2_ catalysts, covering support morphology control, promoter addition, and loading method optimization. For example, Vasiliades et al. [22] prepared Ni-based catalysts supported on CeO_2_ nanorods (CeO_2_-NR) and CeO_2_ nanopolyhedra (CeO_2_-NP). Their comparison showed that Ni/CeO_2_-NR exhibited stronger coke resistance because the CeO_2_ bulk phase had better oxygen migration ability, which increased the carbon deposition gasification rate during CO formation. Daza et al. [23] modified Ni/Mg–Al catalysts with different Ce contents (0, 1, 3, 5, 10 wt.%), and the results showed that Ce doping could effectively suppress carbon deposition. The catalyst modified with 3 wt.% Ce achieved the highest CH_4_ and CO_2_ conversions in the DRM reaction and remained stable for up to 100 h.

Among various structure types, hollow sphere structures have attracted considerable attention due to their high specific surface area and unique confinement effect. Studies have shown that hollow sphere CeO_2_ has already demonstrated certain advantages in the catalysis field. Xie et al. [24] successfully prepared CeO_2_ hollow nanospheres (CeO_2_-HS) via a hydrothermal method for efficient catalytic oxidation of toluene. The excellent performance of the CeO_2_-HS catalyst was mainly attributed to its unique hollow nanosphere structure, which provided a high specific surface area and large pore volume. The CeO_2_-HS catalyst effectively promoted the conversion of these intermediates and facilitated the cleavage of the aromatic ring, ultimately achieving complete mineralization of toluene to CO_2_ and H_2_O. Zhang et al. [25] prepared a copper-based catalyst (Cu-S-Ce) with highly dispersed Cu on hollow sphere CeO_2_ (S-Ce) for the reverse water–gas shift (RWGS) reaction. Their experiments proved that S-Ce played a key role in stabilizing Cu nanoparticles. The large void space inside the hollow sphere significantly enhanced mass transfer, increased the concentration of reactants and active sites, and promoted the formation of oxygen vacancies, thus leading to stable conversion of intermediates and the formation of active sites in the RWGS reaction.

However, most studies on hollow sphere catalysts have been limited to preparing structures with a single shell thickness. In this work, hollow sphere CeO_2_-supported Ni catalysts with different shell thicknesses were prepared by a hydrothermal strategy and an incipient wetness impregnation method, aiming to increase the specific surface area of the CeO_2_ support and achieve uniform dispersion of Ni particles. The catalytic activity and stability of the catalysts were evaluated through DRM performance tests, and the changes in physicochemical properties before and after the reaction were studied in detail, especially the resistance to coke and metal sintering. In addition, temperature-programmed surface reaction and in situ diffuse reflectance spectroscopy were used to analyze the activation behavior of CH_4_ and CO_2_ on the Ni-based catalysts and the possible reaction pathways and mechanism.

## 2. Materials and Methods

### 2.1. Catalyst Preparation

SiO_2_ template microspheres were synthesized by the classic Stöber method. The specific procedure was as follows: Amounts of 92 mL of absolute ethanol, 10 mL of ammonia water, and 5 mL of tetraethyl orthosilicate (TEOS, AR) were sequentially added into a beaker and stirred at room temperature for 4 h. After the reaction, the precipitate was collected by centrifugation, washed several times with deionized water and ethanol, and then dried in an oven at 60 °C for 12 h to obtain SiO_2_ microspheres.

A total of 0.15 g of the as-prepared SiO_2_ microspheres was dispersed in 30 mL of absolute ethanol and treated by ultrasonication to obtain a uniform slurry. Meanwhile, cerium nitrate hexahydrate (Ce(NO_3_)_3_·6H_2_O, AR) (4 mmol, 6 mmol, 8 mmol, and 10 mmol) and 0.5 g of urea were dissolved in 40 mL of deionized water, stirred evenly, and then mixed with the SiO_2_ slurry, followed by ultrasonication for 1 h. The resulting mixture was transferred into a stainless-steel autoclave lined with polytetrafluoroethylene and subjected to a hydrothermal reaction at 160 °C for 8 h. After the autoclave was naturally cooled to room temperature, the solid product was collected by centrifugation, washed several times with deionized water and ethanol, and dried in a vacuum oven at 60 °C for 12 h to obtain the CeO_2_@SiO_2_ core–shell precursor. Subsequently, the precursor was placed in a 0.5 M sodium hydroxide (NaOH, AR) solution and etched at 80 °C for 6 h to completely remove the SiO_2_ template. After etching, the solid was collected by centrifugation, washed repeatedly with deionized water and ethanol until clean, and dried in a vacuum oven (DGG-9036AD (Qingdao Lanten Science and Education Instrument Equipment Co., Ltd., Qingdao, China)) at 60 °C for 12 h. Finally, the product was calcined in a muffle furnace from room temperature to 500 °C at a heating rate of 2 °C min^−1^ and held for 4 h to obtain a series of hollow CeO_2_ sphere supports, which were denoted as *x*CeO_2_-H (where *x* represents the millimolar amount of added Ce(NO_3_)_3_·6H_2_O, with values of 6, 8, and 10).

The bulk CeO_2_ was prepared by a direct calcination of a certain amount of Ce(NO_3_)_3_·6H_2_O at 500 °C for 5 h, which was labeled as CeO_2_.

The Ni/*x*CeO_2_-H series and Ni/CeO_2_ catalysts were prepared by an incipient wetness impregnation method. The specific procedure was as follows: an appropriate amount of nickel nitrate (Ni(NO_3_)_2_·6H_2_O, AR) was dissolved in a certain volume of deionized water to prepare an impregnation solution. Then, 1.00 g of support was added, and the mixture was treated by ultrasonication to achieve uniform dispersion. The resulting mixture was dried in an oven at 60 °C for 12 h, then transferred to a muffle furnace and calcined from room temperature to 500 °C at a heating rate of 2 °C min^−1^ for 4 h. The target Ni-loaded hollow CeO_2_ microsphere catalysts were finally obtained, denoted as Ni/6CeO_2_-H, Ni/8CeO_2_-H, Ni/10CeO_2_-H and Ni/CeO_2_ respectively.

The theoretical Ni loading amount in all catalysts was 10 wt.%.

### 2.2. Catalyst Characterizations

The prepared catalysts were characterized using a variety of techniques, including X-ray diffraction (XRD), Raman spectroscopy, scanning electron microscopy (SEM), thermogravimetric analysis (TGA), X-ray photoelectron spectroscopy (XPS), temperature-programmed reduction of hydrogen (H_2_-TPR), temperature-programmed desorption of carbon dioxide (CO_2_-TPD), temperature-programmed surface reaction of carbon dioxide (CO_2_-TPSR), and temperature-programmed surface reaction of methane (CH_4_-TPSR). The reaction mechanism and the types of reaction intermediates in the catalytic reaction were explored by in situ diffuse reflectance infrared Fourier transform (DRIFT) spectroscopy. More characterization details are provided in the Supplementary Material.

### 2.3. Catalytic Evaluation

An amount of 20 mg of catalyst particles (40–60 mesh) was secured within a U-shaped quartz fixed-bed reactor. Prior to the catalytic reaction, the catalyst was pre-reduced under a flow of 40 mL min^−1^ of 10 vol.% H_2_/Ar gas mixture at 750 °C for 1 h. Subsequently, a reaction feed gas mixture (30 vol.% CH_4_/30 vol.% CO_2_/40 vol.% N_2_) with a total flow rate of 50 mL min^−1^ was introduced, corresponding to a weight hourly space velocity (WHSV) of 150,000 mL·g^−1^·h^−1^. The catalytic activity was evaluated by decreasing the temperature from 750 to 600 °C at intervals of 50 °C. Additionally, the long-term stability of the catalyst was assessed via a continuous DRM reaction at 750 °C for 50 h. The exiting reactants (CH_4_/CO_2_) and products (H_2_/CO) were separated by a packed column (TDX-01) and subsequently analyzed using an online gas chromatograph (GC) (9790II (Zhejiang Fuli Analytical Instruments Co., Ltd., Taizhou, China)) equipped with a thermal conductivity detector (TCD). Using N_2_ as the reference gas, the following equations are employed to calculate the conversion of CH_4_ ($X_{{CH}_{4}}$) and CO_2_ ($X_{{CO}_{2}}$), as well as the molar ratio of H_2_ to CO ($H_{2}/CO$).

(1) $$X_{{CH}_{4}}=\frac{F_{{in\;CH}_{4}}-F_{{out\;CH}_{4}}}{F_{{in\;CH}_{4}}}\times 100\%$$

(2) $$X_{{CO}_{2}}=\frac{F_{{in\;CO}_{2}}-F_{{out\;CO}_{2}}}{F_{{in\;CO}_{2}}}\times 100\%$$

(3) $$H_{2}/CO=\frac{F_{{out\;H}_{2}}}{F_{out\;CO}}\times 100\%$$

where $F_{in}$ represents the molar flow rate of a component in the feed gas, while $F_{out}$ denotes the molar flow rate of the same component in the effluent gas.

## 3. Results

### 3.1. Physicochemical Characterization of Catalysts Before Reaction

#### 3.1.1. Surface Morphology and Microstructure

**Figure 1:**
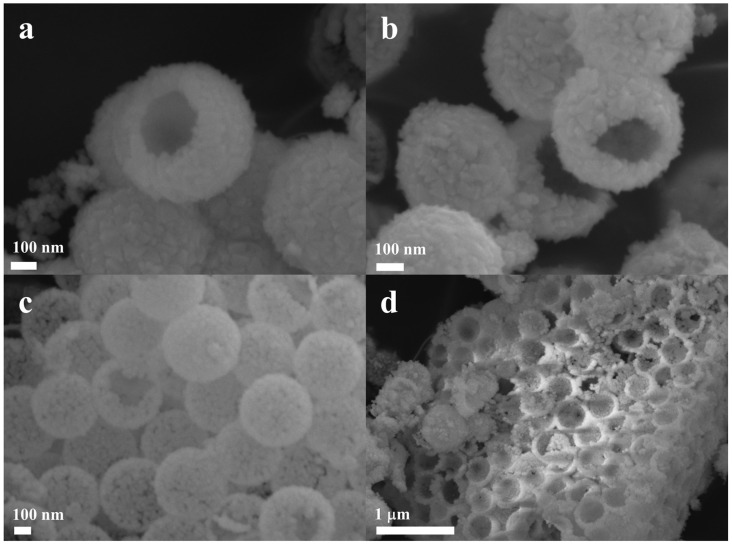
SEM images of (**a**) Ni/10CeO_2_-H, (**b**) Ni/8CeO_2_-H, (**c**) Ni/6CeO_2_-H, (**d**) Ni/4CeO_2_-H catalysts.

The surface morphologies and microstructures of Ni/10CeO_2_-H, Ni/8CeO_2_-H, Ni/6CeO_2_-H, and Ni/4CeO_2_-H catalysts were examined by SEM. As shown in Figure 1, Ni/10CeO_2_-H, Ni/8CeO_2_-H, and Ni/6CeO_2_-H all exhibit well-defined spherical hollow architectures with progressively decreasing shell thickness in the order of Ni/10CeO_2_-H > Ni/8CeO_2_-H > Ni/6CeO_2_-H. Evidently, reducing the amount of Ce(NO_3_)_3_·6H_2_O precursor leads to thinner shell walls and larger internal cavities in the resulting CeO_2_ hollow spheres. Notably, the surface of Ni/6CeO_2_-H is rich in micropores, indicating a higher specific surface area and a greater number of accessible active sites. In contrast, Ni/4CeO_2_-H displays numerous fragmented and collapsed structures, which can be attributed to the insufficient Ce(NO_3_)_3_·6H_2_O content that compromises the structural stability of the hollow spheres during their formation, ultimately resulting in severe structural collapse.

**Figure 2:**
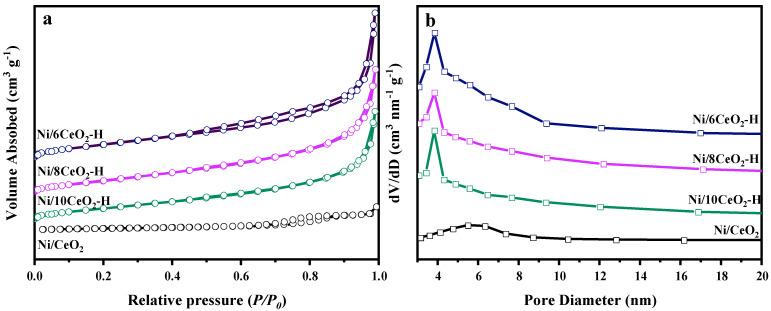
(**a**) Adsorption–desorption isotherms and (**b**) Pore size distributions of Ni/6CeO_2_-H, Ni/8CeO_2_-H, Ni/10CeO_2_-H and Ni/CeO_2_ catalysts.

Figure 2 presents the N_2_ adsorption–desorption isotherms and pore size distribution curves of the as-prepared catalysts. All hollow sphere catalysts exhibited Type IV isotherms, confirming their mesoporous nature. Notably, the hysteresis loops varied among the catalysts: Ni/6CeO_2_-H displayed a pronounced hysteresis loop over the *p*/*p*_0_ range of 0.4–1.0, while Ni/8CeO_2_-H and Ni/10CeO_2_-H showed hysteresis loops in the higher relative pressure range of 0.8–1.0. The broad hysteresis loop observed for Ni/6CeO_2_-H is characteristic of an H3-type loop, which is typically associated with slit-shaped pores formed by the stacking of plate-like particles [26]. In contrast, the Ni/CeO_2_ catalyst exhibited a typical Type II isotherm with an H1-type hysteresis loop, indicative of uniform cylindrical mesopores [27]. The pore size distribution curves shown in Figure 2b further confirm that all three hollow sphere catalysts possess well-defined mesoporous structures. The specific surface area, total pore volume, and average pore diameter of each catalyst are summarized in Table 1. Both the specific surface area and total pore volume follow the order Ni/6CeO_2_-H > Ni/8CeO_2_-H > Ni/10CeO_2_-H > Ni/CeO_2_. Specifically, Ni/6CeO_2_-H achieves a higher specific surface area of 144.7 m^2^ g^−1^, whereas Ni/CeO_2_ exhibits the lowest value of 81.6 m^2^ g^−1^. This trend can be rationalized as follows: as the Ce precursor content increases, the shell thickness of the hollow spheres increases accordingly. Meanwhile, the higher Ce precursor concentration leads to a faster crystal growth rate, resulting in larger nanocrystallite sizes. Since the shell is constructed by the assembly of these nanocrystals, larger building blocks give rise to larger pore diameters. Although the individual pore size increases, the total number of mesopores decreases, leading to a concurrent reduction in both pore volume and specific surface area with increasing Ce precursor content. Consequently, Ni/6CeO_2_-H, with its superior specific surface area, provides a higher density of accessible active sites, thereby facilitating reactant transport and enhancing the contact between reactant molecules and active sites [26,28].

#### 3.1.2. Redox and Basicity Properties

**Figure 3:**
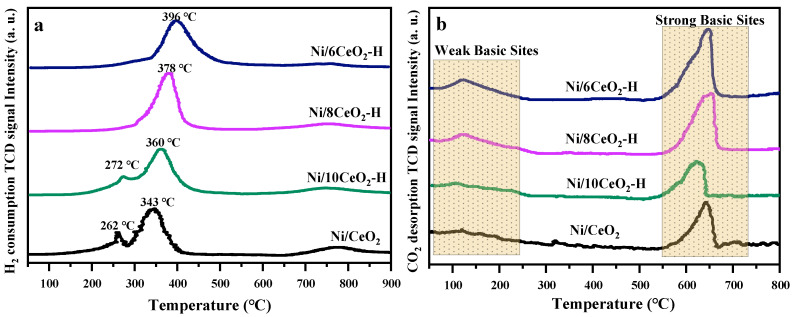
(**a**) H_2_−TPR and (**b**) CO_2_−TPD profiles of Ni/6CeO_2_-H, Ni/8CeO_2_-H, Ni/10CeO_2_-H and Ni/CeO_2_ catalysts.

The H_2_-TPR profiles of the as-prepared catalysts are presented in Figure 3a. It can be observed that Ni/10CeO_2_-H and Ni/CeO_2_ exhibit a distinct reduction peak at 272 and 262 °C, respectively, which is attributed to the reduction of NiO particles with weak interaction with the CeO_2_ support [27]. It is speculated that the low dispersion of Ni species may weaken the Ni–CeO_2_ interaction, leading to the formation of large-sized NiO particles. Notably, no reduction peak is observed in the H_2_-TPR profile of Ni/6CeO_2_-H within the investigated temperature range, which strongly confirms the homogeneous dispersion of NiO within the CeO_2_ support and the presence of strong internal chemical interactions between them. Furthermore, each catalyst displays a prominent reduction peak at approximately 396, 378, 360, and 343 °C for Ni/6CeO_2_-H, Ni/8CeO_2_-H, Ni/10CeO_2_-H, and Ni/CeO_2_, respectively, originating from the co-reduction of CeO_2_ and NiO species [29]. Compared with Ni/8CeO_2_-H, Ni/10CeO_2_-H, and Ni/CeO_2_, the reduction peak of Ni/6CeO_2_-H shifts to a higher temperature, indicating the presence of strong metal–support interactions. This robust MSI effectively suppresses the agglomeration of Ni particles and promotes their uniform dispersion. Overall, these findings further validate the existence of strong Ni–CeO_2_ interactions in the hollow sphere-structured catalysts. In addition, all four catalysts show a noticeable reduction peak within the range of 750–800 °C, which originates from the reduction of bulk lattice oxygen in CeO_2_ [30].

The CO_2_-TPD profiles of the catalysts shown in Figure 3b typically reveal three distinct desorption regions: the low-temperature zone (ca. 200 °C) corresponds to weak Brønsted basic sites, the medium-temperature zone (ca. 400 °C) is associated with moderate-strength Lewis basic sites, and the high-temperature zone (ca. 600 °C) reflects strong basic sites [21,31]. Experimental results demonstrate that all catalysts exhibit significant CO_2_ desorption signals in the high-temperature region, confirming the presence of strong basic sites. Notably, the CO_2_ desorption peak at approximately 120 °C in the low-temperature region becomes increasingly intense as the CeO_2_ shell thickness decreases, which is attributed to weak Brønsted basic sites formed by surface–OH groups [31]. In contrast, Ni/CeO_2_ shows no apparent desorption signal in this temperature range. This discrepancy indicates that the hollow sphere structure significantly modulates the distribution of basic sites on CeO_2_, generating abundant weak basic sites. In the DRM reaction, CH_4_ preferentially reacts with reactive carbon species formed by adsorbed CO_2_ on weak basic sites, thereby effectively suppressing coke deposition and enhancing catalytic stability [32,33]. The CO_2_-TPD results are in good agreement with previous characterization data on coking resistance (e.g., TGA and Raman spectroscopy), providing consistent evidence for the structural advantages of hollow sphere catalysts in mitigating coke formation.

### 3.2. Physicochemical Characterization of Catalysts After Reaction

#### 3.2.1. Catalytic Evaluation

**Figure 4:**
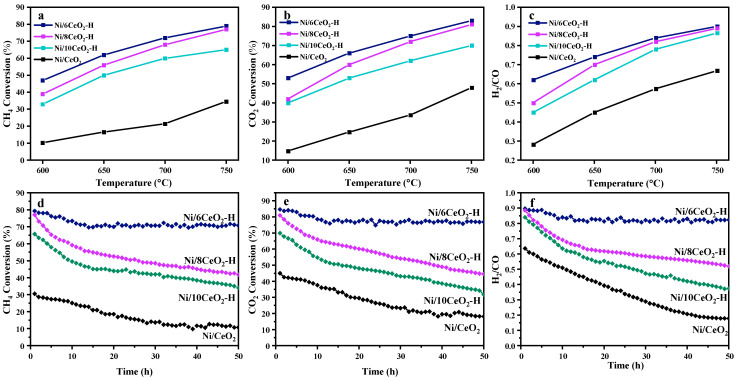
(**a**) CH_4_ conversion, (**b**) CO_2_ conversion, (**c**) molar ratio of H_2_/CO versus reaction temperatures over Ni/6CeO_2_-H, Ni/8CeO_2_-H, Ni/10CeO_2_-H and Ni/CeO_2_ catalysts; (**d**) CH_4_ conversion, (**e**) CO_2_ conversion, and (**f**) molar H_2_/CO ratio as a function of reaction time over Ni/6CeO_2_-H, Ni/8CeO_2_-H, Ni/10CeO_2_-H and Ni/CeO_2_ catalysts at 750 °C.

As shown in Figure 4a–c, within the temperature range of 650–750 °C, all catalysts exhibited a consistent activity trend, Ni/6CeO_2_-H > Ni/8CeO_2_-H > Ni/10CeO_2_-H > Ni/CeO_2_, as reflected by CH_4_ conversion, CO_2_ conversion, and the H_2_/CO molar ratio. These results clearly demonstrate that the hollow spherical structure of the CeO_2_ support significantly enhances CH_4_ and CO_2_ conversion in the DRM reaction. Furthermore, when comparing catalysts supported on hollow CeO_2_ spheres with different shell thicknesses, thinner shells yielded higher catalytic activity. This can be attributed to the larger specific surface area of thinner CeO_2_ shells, which promotes higher dispersion of Ni species, exposes more active sites, and shortens reactant diffusion pathways, thereby improving low-temperature activation capability.

As shown in Figure 4d–f, the four catalysts—Ni/6CeO_2_-H, Ni/8CeO_2_-H, Ni/10CeO_2_-H, and Ni/CeO_2_—displayed markedly distinct catalytic behaviors in terms of CH_4_ and CO_2_ conversions and H_2_/CO molar ratio during the 50 h long-term stability test at 750 °C. Specifically, the CH_4_ conversion, CO_2_ conversion, and H_2_/CO molar ratio over Ni/6CeO_2_-H gradually approached steady values, indicating reasonable operational stability. Ultimately, the CH_4_ conversion, CO_2_ conversion, and H_2_/CO molar ratio over Ni/6CeO_2_-H stabilized at 71%, 76%, and 0.81, respectively. In contrast, the CH_4_ and CO_2_ conversions and H_2_/CO molar ratios over Ni/8CeO_2_-H, Ni/10CeO_2_-H, and Ni/CeO_2_ decreased continuously with time without reaching a stable plateau. The persistent decline in both CH_4_ and CO_2_ conversions clearly indicates severe catalyst deactivation for these catalysts. Furthermore, as shown in Figure 4f, the H_2_/CO molar ratio over Ni/CeO_2_ decreased rapidly with time, which may be attributed to the increasing CO content and decreasing H_2_ content in the products. This trend is likely associated with the occurrence of the RWGS reaction (CO_2_ + H_2_ → CO + H_2_O) [34]. Concurrently, the Boudouard reaction (C + CO_2_ → 2CO) may oxidize surface carbon deposits, further contributing to CO formation [35].

Taken together, these results allow a reasonable inference that the four catalysts differ significantly in their ability to activate CO_2_ and CH_4_, following the order Ni/6CeO_2_-H > Ni/8CeO_2_-H > Ni/10CeO_2_-H > Ni/CeO_2_. Moreover, catalyst deactivation is likely closely associated with changes in physicochemical properties, such as agglomeration of surface Ni particles and coverage of active sites by carbon deposits, which will be investigated in detail in the following sections.

#### 3.2.2. Carbon Deposition Analysis of Spent Catalysts

**Figure 5:**
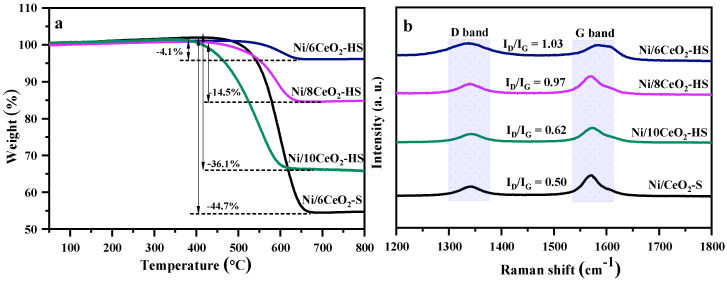
(**a**) TGA curves and (**b**) Raman spectra of the spent catalysts after long−term catalytic stability test.

TGA was performed on four spent catalysts after the 50 h long-term stability test, denoted as Ni/6CeO_2_-HS, Ni/8CeO_2_-HS, Ni/10CeO_2_-HS, and Ni/CeO_2_-S, respectively. The extent of coke deposition was evaluated by continuously monitoring the mass variations during thermal treatment. As shown in Figure 5a, all spent catalysts exhibit distinct mass loss in the temperature range of 400–800 °C, which is primarily attributed to the oxidation of carbonaceous deposits on the catalyst surface. Notably, the weight loss percentages of Ni/6CeO_2_-HS, Ni/8CeO_2_-HS, Ni/10CeO_2_-HS, and Ni/CeO_2_-S within 400–800 °C are approximately 4.1%, 14.5%, 36.1%, and 44.7%, respectively. Evidently, Ni/6CeO_2_-HS exhibits the lowest weight loss, strongly indicating its exceptional coke resistance in the DRM reaction. In contrast, Ni/CeO_2_-S shows the highest weight loss, implying that a substantial amount of coke was deposited on the catalyst surface during the reaction. This result directly accounts for the severe deactivation observed in the long-term stability test.

Raman spectroscopy was further employed to investigate the nature of surface coke species on the spent catalysts. As illustrated in Figure 5b, two characteristic peaks appear at 1340 and 1570 cm^−1^ for all spent catalysts, which are typically assigned to the D band and G band of carbonaceous deposits, respectively. Generally, the D band originates from defects, structural distortions, and amorphous carbon domains containing C–C (sp^3^) or C=C (sp^2^) bonds, whereas the G band is mainly derived from graphitic carbon with the stretching vibration of C=C (sp^2^) bonds [36]. It has been well established that the graphitization degree of surface coke can be evaluated based on the relative intensity ratio of the D band to the G band (I_D_/I_G_) [37]. Herein, the I_D_/I_G_ values over Ni/6CeO_2_-HS, Ni/8CeO_2_-HS, Ni/10CeO_2_-HS, and Ni/CeO_2_-S are estimated to be 1.03, 0.97, 0.62, and 0.50, respectively, indicating that the graphitization degree of surface coke increases in the order of Ni/6CeO_2_-HS < Ni/8CeO_2_-HS < Ni/10CeO_2_-HS < Ni/CeO_2_-S. Clearly, the lowest graphitization degree of coke over Ni/6CeO_2_-HS further confirms its superior coke resistance in the DRM reaction.

#### 3.2.3. Crystalline Phases

**Figure 6:**
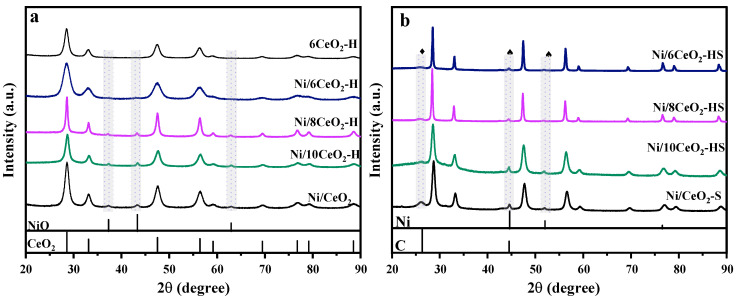
XRD patterns of (**a**) fresh and (**b**) spent catalysts.

The XRD patterns of the fresh and spent catalysts are presented in Figure 6. As shown in Figure 6a, all fresh catalysts of Ni/6CeO_2_-H, Ni/8CeO_2_-H, Ni/10CeO_2_-H, and Ni/CeO_2_ exhibit distinct diffraction peaks at 2*θ* values of 28.5°, 33.0°, 47.5°, 56.3°, 59.0°, 69.4°, 76.7°, 79.0°, and 88.4°, which can be respectively indexed to the (111), (200), (220), (311), (222), (400), (311), (420), and (422) planes of the fluorite-structured CeO_2_ support (JCPDS PDF#43-1002) [38]. In addition, Ni/8CeO_2_-H, Ni/10CeO_2_-H, and Ni/CeO_2_ display characteristic diffraction peaks at 2*θ* = 37.2°, 43.3°, and 62.9°, corresponding to the (111), (200), and (220) planes of NiO (JCPDS PDF#47-1049), respectively [38]. Notably, no distinguishable NiO diffraction peaks were detected over the Ni/6CeO_2_-H catalyst. To eliminate the possibility that the undetectable NiO signals stem from partial incorporation of Ni into the CeO_2_ lattice, we compared the XRD patterns of Ni/6CeO_2_-H with those of the pure 6CeO_2_-H support. No shifts in the CeO_2_ diffraction peaks were observed for Ni/6CeO_2_-H, demonstrating that Ni species are highly dispersed as ultrafine nanoparticles on the hollow spherical support, thereby weakening the NiO diffraction signals below the detection limit. In contrast, the NiO particles in the other three catalysts are predominantly present as larger aggregates on the external surface, giving rise to clearly resolvable diffraction peaks.

Figure 6b reveals the XRD patterns of the spent catalysts. Additional diffraction peaks at 2*θ* = 26.2° and 44.4°, attributable to surface-deposited carbon species (JCPDS PDF#75-1621) [39], are observed for Ni/8CeO_2_-HS, Ni/10CeO_2_-HS, and Ni/CeO_2_-S, with the carbon peak at 26.2° being particularly intense. In contrast, such carbon-related peaks are barely detectable for Ni/6CeO_2_-HS. These observations indicate that significant coke deposition occurs on the surfaces of Ni/8CeO_2_-HS, Ni/10CeO_2_-HS, and Ni/CeO_2_-S, whereas Ni/6CeO_2_-HS maintains a markedly lower content of coke deposition. This finding further corroborates the superior coke resistance of Ni/6CeO_2_-H in the DRM reaction and is in excellent agreement with the TGA and Raman spectroscopy results. Moreover, Figure 6b also displays the characteristic diffraction peaks of metallic Ni on the spent catalysts. Peaks at 2*θ* = 44.5°, 51.8°, and 76.4° correspond to the (111), (200), and (220) planes of metallic Ni (JCPDS PDF#87-0712), respectively [40]. The peak intensities assignable to metallic Ni over Ni/8CeO_2_-HS, Ni/10CeO_2_-HS, and Ni/CeO_2_-S are substantially higher than those over Ni/6CeO_2_-HS, indicating that severe metal sintering has occurred on the surface of the thick-shelled hollow spherical and bulk CeO_2_-supported catalysts during the DRM reaction. In contrast, the strong chemical interaction between Ni and CeO_2_ in Ni/6CeO_2_-H effectively anchors the surface Ni particles, suppressing their agglomeration at elevated temperatures and thereby inhibiting metallic Ni sintering.

#### 3.2.4. Morphological Evolution of Spent Catalysts

**Figure 7:**
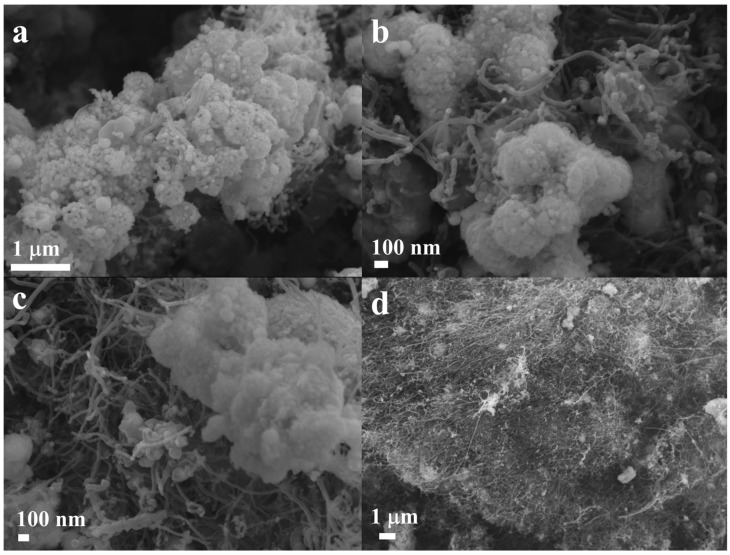
SEM images of (**a**) Ni/6CeO_2_-HS, (**b**) Ni/8CeO_2_-HS, (**c**) Ni/10CeO_2_-HS, (**d**) Ni/CeO_2_-S catalysts.

SEM analysis was performed on the catalysts after 50 h of reaction. As shown in Figure 7, the surface of Ni/6CeO_2_-HS is largely free of coke deposition, whereas extensive carbon accumulation is observed on Ni/8CeO_2_-HS and Ni/10CeO_2_-HS. Notably, the surface of Ni/CeO_2_-S is completely engulfed by coke, which accounts for its rapid deactivation observed in the stability test. In contrast, the absence of coke coverage on Ni/6CeO_2_-HS allows the Ni particles to preserve their intrinsic activity, thereby endowing this catalyst with superior catalytic stability. These SEM observations are in excellent agreement with the TGA and Raman results, providing direct visual evidence of the distinct coke contents on the spent catalysts. The remarkable coke resistance of Ni/6CeO_2_-HS can be ascribed to the strong metal–support interaction between Ni and CeO_2_, which effectively suppresses the agglomeration and growth of carbonaceous species during the reaction.

#### 3.2.5. Surface Valance States of Ni and Ce

**Figure 8:**
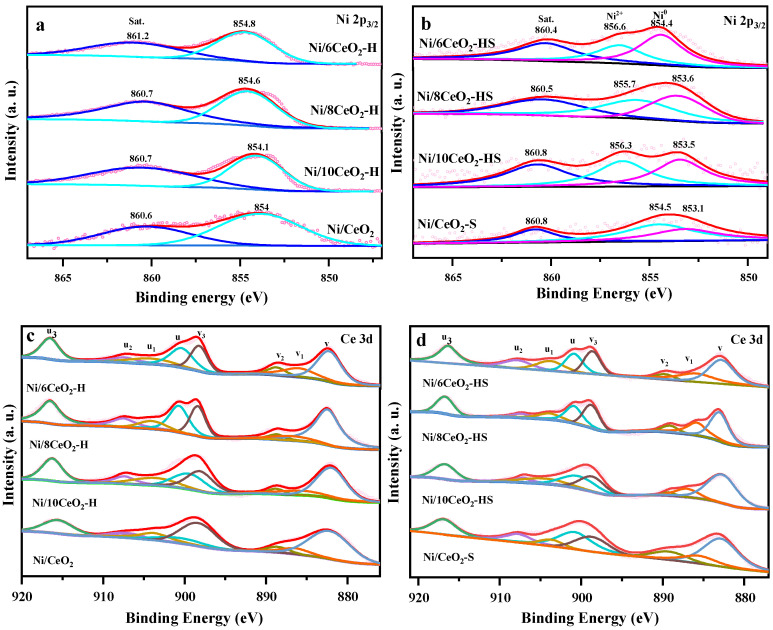
XPS spectra of Ni 2p_3/2_ and Ce 3d for fresh and spent catalysts: (**a**) Ni 2p_3/2_ of fresh catalyst, (**b**) Ni 2p_3/2_ of spent catalyst, (**c**) Ce 3d of fresh catalyst, (**d**) Ce 3d of spent catalyst.

The chemical valence states of Ni and Ce on both fresh and spent catalysts were investigated by XPS. In the Ni 2p_3/2_ spectra of the fresh catalysts shown in Figure 8a, two characteristic peaks attributed to Ni^2+^ are observed, with the main peak and satellite peak located at 854.1–854.8 eV and 860.6–861.2 eV, respectively. The binding energies of Ni/6CeO_2_-H, Ni/8CeO_2_-H, Ni/10CeO_2_-H, and Ni/CeO_2_ are 854.8, 854.6, 854.1, and 854.0 eV, respectively. Notably, catalysts with thinner shells exhibit higher binding energies, indicating stronger metal–support interactions [41]. In the Ni 2p_3/2_ spectra of the spent catalysts presented in Figure 7b, an additional peak corresponding to metallic Ni^0^ emerges in the range of 853.1–854.4 eV. Since metallic Ni is widely recognized as the active center for CH_4_ adsorption and activation, the proportion of Ni^0^ species is closely correlated with the initial CH_4_ conversion. According to Silva et al. [42,43], increasing the electron density at Ni^0^ active sites enables electron transfer from Ni^0^ to the antibonding orbitals of the C–H bonds in CH_4_, thereby effectively promoting methane dissociation and inducing C–H bond cleavage. To further verify the relationship between reducibility and shell thickness, the ratio of Ni^0^/(Ni^0^ + Ni^2+^) was calculated [44]. As summarized in Table 2, the Ni^0^/(Ni^0^ + Ni^2+^) ratios for Ni/6CeO_2_-HS, Ni/8CeO_2_-HS, Ni/10CeO_2_-HS, and Ni/CeO_2_ are 0.63, 0.60, 0.48, and 0.40, respectively. The higher Ni^0^ density on Ni/6CeO_2_-HS indicates a greater number of accessible active sites, which accounts for its enhanced catalytic activity.

In the Ce 3d XPS spectra of all catalysts shown in Figure 8c,d, several characteristic multiplet splitting patterns are observed, which can be attributed to Ce^3+^ (u_1_, v_1_) and Ce^4+^ (u, u_2_, u_3_, v, v_2_, v_3_) species [45,46]. The proportion of Ce^3+^ relative to the total Ce^3+^ and Ce^4+^ can be estimated by calculating the ratio of the peak area assignable to Ce^3+^ to the total peak area of the Ce 3d spectra, at listed in Table 2. The fresh catalysts exhibit Ce^3+^/(Ce^3+^ + Ce^4+^) ratios in the order of Ni/6CeO_2_-H > Ni/8CeO_2_-H > Ni/10CeO_2_-H > Ni/CeO_2_, indicating a higher surface concentration of Ce^3+^ species on Ni/6CeO_2_-H. Additionally, the spent catalysts display distinct redox behaviors under reaction conditions. After the DRM reaction, the Ce^3+^/(Ce^3+^ + Ce^4+^) ratio of Ni/6CeO_2_-HS decreases from 0.35 to 0.21, representing the most pronounced reduction among all catalysts. The ratios for Ni/8CeO_2_-H, Ni/10CeO_2_-H, and Ni/CeO_2_ decrease from 0.23 to 0.17, from 0.18 to 0.14, and from 0.17 to 0.15, respectively. These variations demonstrate that the Ce species in Ni/6CeO_2_-H participate more extensively in the redox cycles under reaction conditions [47].

Notably, two quantitative XPS results clearly verify this enhanced synergism. First, fresh Ni/6CeO_2_-H delivers the highest Ni 2p_3/2_ binding energy of 854.8 eV, revealing the strongest electron transfer from Ni cations to the ceria support. Second, it also owns the maximum Ce^3+^/(Ce^3+^ + Ce^4+^) ratio of 0.35, corresponding to abundant surface oxygen vacancies originating from Ni–Ce electronic exchange. After DRM reaction, Ni/6CeO_2_-H exhibits the most dramatic decrease in Ce3+ proportion and the largest Ni^0^/(Ni^0^ + Ni^2+^) value of 0.63, which confirms extensive Ce-involved redox cycles and abundant stabilized metallic Ni sites under working conditions. Combined analysis of the Ni 2p_3/2_ and Ce 3d XPS data reveals that Ni/6CeO_2_-H exhibits superior Ni–Ce synergistic effects compared to the other three catalysts.

### 3.3. Surface Reaction Process by TPSR

**Figure 9:**
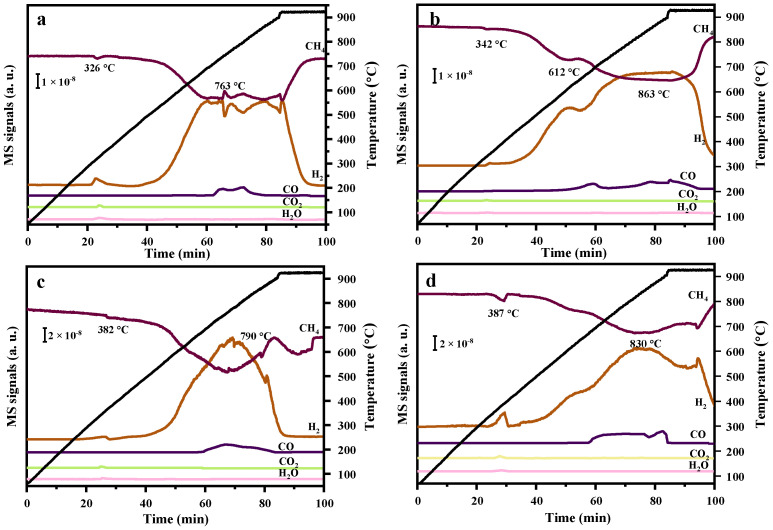
CH_4_−TPSR profiles of (**a**) Ni/6CeO_2_-H, (**b**) Ni/8CeO_2_-H, (**c**) Ni/10CeO_2_-H and (**d**) Ni/CeO_2_ catalysts.

The CH_4_-TPSR (temperature-programmed surface reaction) profiles of Ni/6CeO_2_-H, Ni/8CeO_2_-H, Ni/10CeO_2_-H, and Ni/CeO_2_ catalysts are presented in Figure 9a–d. Real-time monitoring of CH_4_ consumption and product signals (H_2_, CO, CO_2_, H_2_O) by mass spectrometry reveals the influence of CeO_2_ hollow sphere shell thickness on CH_4_ activation, dissociation, and carbon gasification over the catalyst surfaces. Distinct CH_4_ consumption peaks are observed at 326, 342, 382, and 387 °C for Ni/6CeO_2_-H, Ni/8CeO_2_-H, Ni/10CeO_2_-H, and Ni/CeO_2_, respectively, accompanied by simultaneous H_2_ generation and synchronous CO_2_ and H_2_O signal peaks [48,49]. These products originate from the oxidation of unstable CH_x_* species, generated by CH_4_ dissociation, by adsorbed oxygen species on the catalyst surface. The onset temperature of CH_4_ consumption directly reflects the catalytic activation capability. The fact that Ni/6CeO_2_-H achieves rapid partial CH_4_ activation at as low as 326 °C indicates that its thin-shell structure endows the catalyst with a higher specific surface area, abundant CeO_2_ oxygen vacancies, and highly dispersed Ni active sites, establishing strong metal–support interactions that significantly lower the energy barrier for C–H bond cleavage [50].

In addition to the low-temperature activation differences, all catalysts exhibit intense H_2_ signals in the high-temperature region at 763, 863, 790, and 830 °C, respectively. Notably, Ni/6CeO_2_-H initiates strong CH_4_ cracking at a relatively low temperature of 763 °C, which is fully consistent with its superior low-temperature performance. This further confirms that the thin-shell design exposes more Ni active sites and CeO_2_ oxygen vacancies owing to the higher specific surface area, thereby promoting deep CH_4_ dissociation. The H_2_ peak of Ni/8CeO_2_-H appears at a higher temperature of 863 °C, while that of Ni/10CeO_2_-H emerges at 790 °C but exhibits a stronger overall high-temperature cracking capability. The intensities of both CH_4_ consumption and H_2_ generation follow the order Ni/6CeO_2_-H < Ni/8CeO_2_-H < Ni/10CeO_2_-H < Ni/CeO_2_ (with signal scales of 1 × 10^−8^ for panels a and b, and 2 × 10^−8^ for panels c and d), indicating that Ni/10CeO_2_-H and Ni/CeO_2_ possess the strongest CH_4_ cracking ability. This can be attributed to the larger Ni particles supported on these catalysts. It has been demonstrated that larger Ni nanoparticles exhibit stronger C–H bond cleavage capability toward CH_4_ at elevated temperatures, generating a greater quantity of CH_x_* species [12]. These CH_x_* species undergo further cracking to produce substantial carbon deposits. Accordingly, during the DRM stability tests, the catalytic activity profiles of Ni/10CeO_2_-H and Ni/CeO_2_ fail to reach a stable plateau and instead decline continuously, indicating that the generated coke cannot be effectively removed. This leads to progressive coverage of surface Ni particles by carbon deposits, ultimately resulting in catalyst deactivation.

**Figure 10:**
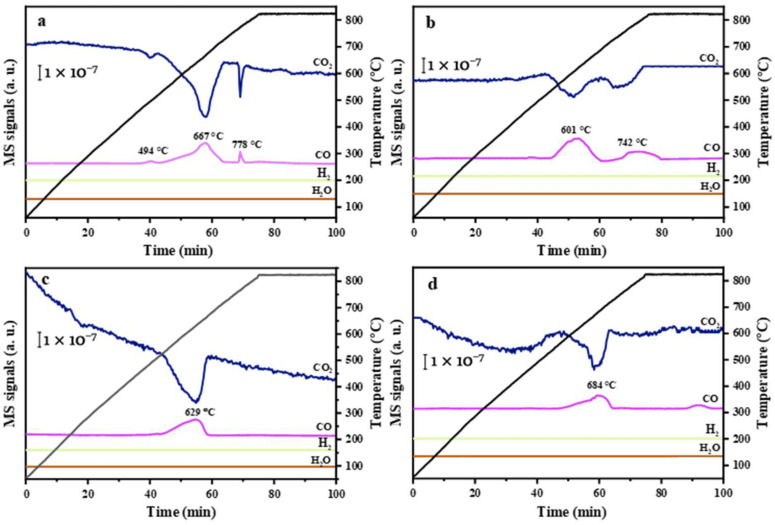
CO_2_−TPSR profiles of (**a**) Ni/6CeO_2_-H, (**b**) Ni/8CeO_2_-H, (**c**) Ni/10CeO_2_-H and (**d**) Ni/CeO_2_ catalysts.

To evaluate the coke removal capability by CO_2_, CO_2_-TPSR analysis was subsequently performed over the catalysts following CH_4_-TPSR tests. Figure 10a–d present the CO_2_-TPSR profiles of Ni/6CeO_2_-H, Ni/8CeO_2_-H, Ni/10CeO_2_-H, and Ni/CeO_2_, respectively. Temperature-programmed heating under pure CO_2_ atmosphere with real-time mass spectrometric monitoring of CO_2_ consumption and product signals (CO, H_2_, H_2_O) reveals the regulatory effect of CeO_2_ hollow sphere shell thickness on CO_2_ activation capability, dissociation efficiency, and oxygen species supply. The CO_2_ consumption in CO_2_-TPSR originates from CO_2_ dissociation and the reaction of CO_2_ with carbonaceous species to produce CO. As the shell thickness increases from thin to thick, the CO_2_ activation performance of the catalysts decreases significantly. Ni/6CeO_2_-H exhibits a distinct CO_2_ signal drop at as low as 429 °C, whereas Ni/8CeO_2_-H, Ni/10CeO_2_-H, and Ni/CeO_2_ show no detectable CO_2_ consumption within this temperature range. This indicates that the thin-shell structure enables effective CO_2_ adsorption and dissociation into CO and reactive O* species at low temperatures, while the thick-shell structure substantially restricts CO_2_ adsorption capacity [51]. Under identical MS signal scales (1 × 10^−7^), Ni/10CeO_2_-H and Ni/CeO_2_ display only a single, weak CO peak at approximately 629 and 684 °C, respectively, with peak intensities far lower than those of the former two catalysts. This demonstrates that the thick-shell structure severely hinders Ni exposure and oxygen vacancy utilization, resulting in the lowest CO_2_-to-CO conversion efficiency. Furthermore, the absence of pronounced CO_2_ consumption peaks suggests that substantial coke deposition occurred during the preceding CH_4_-TPSR test, which further compromises the CO_2_ activation capability of Ni/10CeO_2_-H and Ni/CeO_2_ [50].

### 3.4. Reaction Mechanism by In Situ DRIFT Spectra

**Figure 11:**
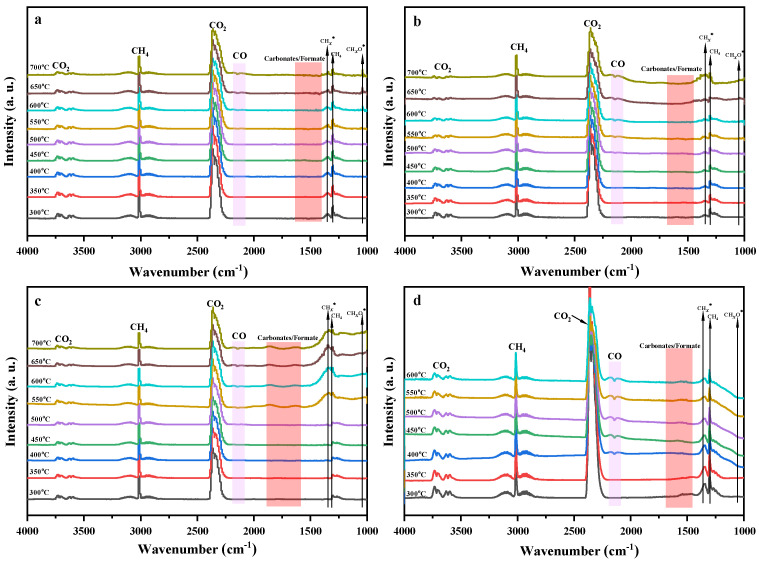
In situ DRIFT spectra of reduced (**a**) Ni/6CeO_2_-H, (**b**) Ni/8CeO_2_-H, (**c**) Ni/10CeO_2_-H and (**d**) Ni/CeO_2_ catalysts.

Figure 11 presents the in situ DRIFT spectra of the as-prepared catalysts under temperature-programmed operation in a CH_4_/CO_2_/Ar feed mixture. The representative DRM reaction pathways along with the intermediates identified in the DRIFT spectra are outlined in Equations (4)–(11). It is noteworthy that Figure 11d cannot provide reliable temperature-dependent IR spectra for subsequent analysis due to severe coke deposition on Ni/CeO_2_ above 600 °C. Various intermediates involved in the DRM reaction are clearly detected in the DRIFT spectra. Specifically, the IR signals assignable to gas-phase CH_4_ (3016/1304 cm^−1^) and gas-phase CO_2_ (3752/3600 and 2240–2400 cm^−1^) gradually weaken with increasing reaction temperature, which is well consistent with the thermodynamic characteristics of the DRM reaction [52,53]. The IR peak at 1540 cm^−1^ corresponds to bicarbonate (HCO_3_*) species formed via the reaction of CO_2_ with –OH (Equation (4)) [54]. The IR peaks in the range of 1520–1400 cm^−1^ indicate the presence of carbonate (CO_3_*) species, likely derived from the reaction of CO_2_ with O* (Equation (5)) [50]. The IR signal at 2120–2180 cm^−1^ serves as a characteristic indicator of gas-phase CO, while the peak at 1346 cm^−1^ is assigned to the asymmetric vibration of CH_x_* species, revealing the formation pathway of CH_x_* and H* from CH_4_ cracking (Equation (6)) [55]. The appearance of HCOO* species, with an IR band at 1600 cm^−1^, may arise from the reactions between HCO_3_*/CO_3_* and H* (Equations (7) and (8)) [54]. In addition, the reaction of CH_x_* with –OH yields CH*_x_*O* species (Equation (9)), giving rise to an IR peak at 1050 cm^−1^. Both HCOO* and CH*_x_*O* are critical unstable intermediates in the DRM reaction, as they rapidly decompose into CO and –OH (Equation (10)) or CO and H* (Equation (11)).

Further observations of Figure 11a reveal that the IR signals of CH_4_ and CO_2_ over the Ni/6CeO_2_-H catalyst undergo significant changes in the temperature range of 300–700 °C. The progressive decrease in the IR signals of CH_4_ and CO_2_ with increasing temperature indicates the high catalytic activity of this catalyst toward CH_4_ and CO_2_ conversion. The IR peak intensities of CH_x_* species over Ni/6CeO_2_-H gradually decrease with rising temperature, whereas the IR peak intensity of CH*_x_*O* exhibits an opposite trend, thereby confirming that CH*_x_*O* species is formed via the reaction of CH_x_* with –OH. Since CH_x_* derived from CH_4_ cracking is regarded as a key precursor for coke deposition, its further cracking leads to surface coke accumulation and subsequent catalyst deactivation. The timely conversion of CH_x_* to CH*_x_*O* over Ni/6CeO_2_-H can effectively reduce the probability of coke formation, thereby enhancing its coke resistance during the reaction. In contrast, as shown in Figure 11b–d, no distinct IR peak attributable to CH*_x_*O* species is observed over Ni/8CeO_2_-H, Ni/10CeO_2_-H, and Ni/CeO_2_, while the IR signals of CH_x_* species become increasingly intense. The CH_x_* species generated during the reaction cannot be effectively consumed, resulting in continuous cracking of CH_x_* into substantial coke deposits and ultimately severe catalyst deactivation.

**Scheme 1:**
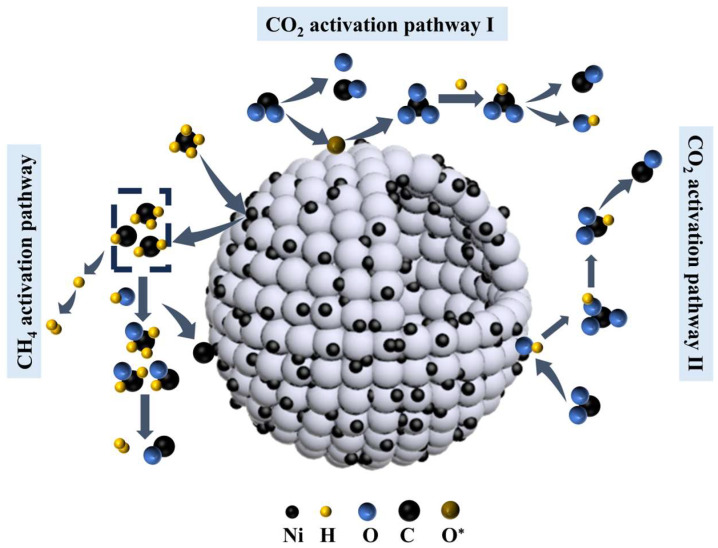
Schematic illustration of a proposed DRM reaction mechanism over reduced Ni/6CeO_2_-H catalysts.

Based on the in situ DRIFTS results, a plausible reaction mechanism for DRM over the reduced Ni/6CeO_2_-H catalyst can be proposed, as illustrated in Scheme 1. The CO_2_ conversion pathway primarily relies on the CeO_2_ support, involving O* to form CO_3_* species and –OH-mediated activation to form HCO_3_* species. The resulting CO_3_* and HCO_3_* intermediates further react with H* to generate unstable HCOO* species, which rapidly decompose into CO and –OH. Meanwhile, CH_4_ activation is predominantly catalyzed by metallic Ni particles, dissociating into CH_x_* fragments and H*. According to the CO_2_-TPD results (Figure 3b), the Ni/CeO_2_ catalyst exhibits insufficient CO_2_ activation capacity, which significantly suppresses the HCOO* formation pathway. The inadequate supply of –OH species hinders the reaction between CH_x_* and –OH to form CH*_x_*O* intermediates, thereby promoting further fragmentation of CH_x_* and the formation of surface carbon deposits. Moreover, the thick-shelled hollow structure and the relatively large Ni particles on the Ni/CeO_2_ surface facilitate graphitization of carbon precursors, exacerbating coke formation, as corroborated by the Raman spectra in Figure 5b [56].

(4) $$CO+{OH}^{*}\to {HCO}_{3}^{*}$$

(5) $${CO}_{2}+O^{*}\to {CO}_{3}^{*}$$

(6) $${CH}_{4}\to {CH}_{x}^{*}+H^{*}$$

(7) $${HCO}_{3}^{*}+H^{*}\to {HCOO}^{*}+{OH}^{*}$$

(8) $${CO}_{3}^{*}+H^{*}\to {HCOO}^{*}+O^{*}$$

(9) $${CH}_{x}^{*}+{OH}^{*}\to CH_{x}O^{*}+H^{*}$$

(10) $${HCOO}^{*}\to {OH}^{*}+CO$$

(11) $$CH_{x}O^{*}\to CO+H^{*}$$

In contrast, the thin-shelled architecture of Ni/6CeO_2_-H substantially enhances CO_2_ activation, thereby generating reactive surface –OH species that readily react with CH_x_* fragments. In this process, the –OH species serve as “coke precursor scavengers,” not only promoting the conversion of CH_x_* to CH*_x_*O but also effectively suppressing coke deposition on Ni/6CeO_2_-H. The strong Ni–Ce interfacial interaction over the reduced Ni/6CeO_2_-H catalyst further facilitates the participation of –OH species in the reaction, accelerating the removal of carbon precursors and inhibiting coke formation.

## 4. Conclusions

CeO_2_ hollow sphere supports were successfully synthesized via a hard template method combined with a hydrothermal approach, with the shell thickness precisely controlled by tuning the precursor feeding ratio. DRM evaluation revealed that the Ni/6CeO_2_-H catalyst exhibited significantly superior catalytic activity and stability compared to Ni/8CeO_2_-H, Ni/10CeO_2_-H, and Ni/CeO_2_. This outstanding performance was primarily attributed to the thin-shell architecture, which endowed the catalyst with a higher specific surface area and pore volume, thereby exposing more Ni active sites. Moreover, the Ni/6CeO_2_-H catalyst possessed excellent CO_2_ activation capability and abundant weak basic sites, facilitating the in situ generation of a large quantity of surface –OH species that effectively gasified and eliminated coke deposits, thus preserving long-term catalytic activity and stability. In situ diffuse reflectance infrared Fourier transform spectroscopy further demonstrated that surface –OH groups served as the critical active species in the DRM reaction. These –OH groups not only participated in CO_2_ activation and its subsequent conversion into carbonate/formate intermediates but also effectively suppressed the further decomposition of CH_x_* species derived from CH_4_ cracking, thereby preventing coke deposition.

## Figures and Tables

**Table 1:** Porous properties of Ni/6CeO_2_-H, Ni/8CeO_2_-H, Ni/10CeO_2_-H and Ni/CeO_2_.

Catalysts	Specific Surface Area ^1^ (m^2^ g^−1^)	Total Pore Volume ^2^ (cm^3^ g^−1^)	Pore Diameter ^2^ (nm)
Ni/6CeO_2_-H	144.7	0.41	4.21
Ni/8CeO_2_-H	105.3	0.34	4.36
Ni/10CeO_2_-H	87.2	0.29	4.51
Ni/CeO_2_	81.6	0.26	6.12

^1^ Calculated by the Brunauer–Emmett–Teller (BET) method. ^2^ Determined by the Barrett–Joyner–Halenda (BJH) method from the desorption branch.

**Table 2:** XPS analysis of Ni 2p3/2 and Ce 3d.

Catalysts	Ni^0^/(Ni^0^ + Ni^2+^)	Ce^3+^/(Ce^4+^ + Ce^3+^)	
Ni/6CeO_2_-H	0.63	0.35	0.21 (S)
Ni/8CeO_2_-H	0.60	0.23	0.17 (S)
Ni/10CeO_2_-H	0.48	0.18	0.14 (S)
Ni/CeO_2_	0.40	0.17	0.15 (S)

## Data Availability

The original contributions presented in this study are included in the article/Supplementary Materials. Further inquiries can be directed to the corresponding author.

## Supplementary Materials

The following supporting information can be downloaded at https://www.mdpi.com/article/10.3390/nano16140868/s1.
