# Supplementary material for: Ni Supported on Hollow CeO2 Microspheres with Controllable Shell Thickness for Catalytic Dry Reforming of Methane
Source: Nanomaterials (Basel). 2026 Jul 15;16(14):868. doi: 10.3390/nano16140868 (PMC13415152; doi:10.3390/nano16140868)
Supplement: Supplementary file 1 [file nanomaterials-16-00868-s001.zip › nanomaterials-4421943-supplementary.pdf]

Supplementary material

Ni Supported on Hollow CeO_2 Microspheres with Controllable Shell Thickness for Catalytic Dry Reforming of Methane

Junyi Liu[a], Hongyu Cui[a], Tianqi Cao[a], Chuanhui Zhang[a,b,] *

[a] Institute of Materials for Energy and Environment, College of Materials Science and Engineering, Qingdao University, Qingdao 266071, China

[b] Faculty of Chemical Engineering and Energy Technology, Shanghai Institute of Technology, Shanghai 201418, China

*** Corresponding author**

Prof. Chuanhui Zhang, E-mail: zhangch@qdu.edu.cn

Catalyst Characterization

Powder X-ray diffraction (XRD) patterns were recorded on a Rigaku Ultima IV diffractometer with Cu Kα (λ = 0.154184 nm) radiation at 40 kV and 40 mA. The images of scanning electron microscopy (SEM) was collected on a JSM-7800F, respectively. Nitrogen adsorption-desorption isotherms were examined on a Quantachrome Autosorb-iQ3 sorptometer after a degassing treatment at 200 °C for 10 h. Thermogravimetric analysis (TGA) was employed using a METTLER TOLEDO TGA 2 to determine the quantity of coke deposition on the spent catalysts. 10 mg of the spent catalyst was heated in an air flow (50 mL min^{-1}) from room temperature to 800 oC at a heating rate of 10 °C min^{-1}. Raman spectra were collected to analyze the structure of the coke deposition on the spent catalysts by a Renishaw in Via plus Micro-Raman spectrometer equipped with a 532 nm laser beam under ambient condition. X-ray photoelectron spectroscopy (XPS) was conducted on PHI 5000 Versaprobe III with a monochromatized Al Kα X-ray source (1486.6 eV). The binding energy (BE) was determined by utilizing C1s of adventitious carbon (284.6 eV) as a reference.

Temperature-programmed reduction of hydrogen (H_2-TPR), temperature-programmed desorption of carbon dioxide (CO_2-TPD) and temperature-programmed surface reaction (TPSR) experiments were performed using a Quantachrome Chembet

Pulsar analyzer coupled with a mass spectrometer (Hiden, HPR20-EGA). The H_2-TPR profile was collected in the range of 50-900 °C under 5% H_2/Ar (30 mL min^{-1}) flow after a pretreatment at 300 °C for 1 h in an argon atmosphere. For CO_2-TPD, the catalyst was pre-reduced in a 5% H_2/Ar (30 mL min^{-1}) atmosphere at 750 °C for 1 h, followed by flushing with Ar (20 mL min^{-1}) and cooling to 50 °C. Then, the atmosphere was switched to CO_2, and the catalyst was allowed to adsorb for 1 h until saturation. Next, the gas was switched back to Ar and maintained at 50 °C for 60 min to remove physically adsorbed CO_2. Once the mass spectrometer baseline stabilized, the catalyst was heated in an Ar atmosphere at a rate of 10 °C min^{-1} from 50-800 °C, and the MS signal of CO_2 (m/z = 44) was recorded. In the CH_4-TPSR experiment, the catalyst was first pre-reduced at 750 °C for 1 h under a 5% H_2/Ar atmosphere in a fixed-bed reactor, then cooled to room temperature, then the catalyst was pre-reduced at 700 °C for 1 h under a 5% H_2/Ar atmosphere at chemisorption apparatus, followed by flushing with Ar gas and cooling down to 50 °C. After switching to a 5% CH_4/Ar (30 mL min^{-1}) atmosphere and waiting for MS baseline stabilization, the catalyst was heated in an Ar atmosphere at a rate of 10 °C min^{-1} from 50-800 °C, and kept at 800 °C for 30 min. During this process, the MS signals of CH_4 (m/z = 16), H_2 (m/z = 2), CO_2 (m/z = 44), CO (m/z = 28), and H_2O (m/z = 18) were recorded. After the CH_4-TPSR test, the catalyst underwent the CO_2-TPSR test. The reactor temperature was first cooled to 50 °C, and the atmosphere was switched to CO_2 (30 mL min^{-1}). Once the mass MS signal stabilization, the catalyst was heated in an Ar atmosphere at a rate of 10 °C min^{-1} from 50-800 °C, and kept at 800 °C for 30 min. The MS signals of H_2 (m/z = 2), CO_2 (m/z = 44), CO (m/z = 28), and H_2O (m/z = 18) were recorded. In situ diffuse reflectance infrared Fourier transform (DRIFT) spectra were recorded on a Thermofisher Scientific Nicolet iS50 spectrometer equipped with a MCT detector. The DRIFT spectra were obtained with a resolution of 8 cm^{-1} and 64 scans. The catalyst was first pre-reduced at 750 °C for 1 h under a 5% H_2/Ar atmosphere in a fixed-bed reactor, then cooled to room temperature. Subsequently, the catalyst was transferred to the IR reaction cell and reduced at 650 °C for 1 h under a 5% H_2/Ar atmosphere. Next, the atmosphere was switched to Ar (8 mL min^{-1}), and the IR background was collected in the temperature

range of 300-700 ºC with each interval of 50 ºC. After background collection, a gas mixture containing CH_4 (6 mL min^{-1}), CO_2 (6 mL min^{-1}) and Ar (8 mL min^{-1}) were introduced to the reaction cell, and DRIFT spectra were collected in the temperature range of 300-700 ºC with each interval of 50 ºC.
